# Supplementary material for: The discovery of novel noncoding RNAs in 50 bacterial genomes
Source: Nucleic Acids Res. 2024 Apr 22;52(9):5152–65. doi: 10.1093/nar/gkae248 (PMC11109978; doi:10.1093/nar/gkae248)
Supplement: gkae248_Supplemental_Files [file gkae248_supplemental_files.zip › Narunsky 50 Genomes RNA Motifs Files.zip!/Publication/38-B_aphidicola/Figures/LRC-38-5-NC_004061_1_IGR_20_523116_523304.sto.pdf]

LRC-38-5-NC_004061_1_IGR_20_523116_523304.sto.r2r

5′-○●AAA●AYAUY●GAURAUCU●A●A●AARRGC●●U○●●CYUUUAUUUURYCAAACGCUU●R●AU●AAAYGUCAAGCRAC●AUUCCRAUAUACUUGRCU●AR●U●UAARUR○○○CUG●AURUU●U●UUAU●AUA●●●UGCAGUUUUUAA○○○●●AUAAAYA●CGGAAAUUCAACUA○AGAGAGAGCUC
